# Fluorescent Chitosan Modified with Heterocyclic Aromatic Dyes

**DOI:** 10.3390/ma14216429

**Published:** 2021-10-26

**Authors:** Halina Kaczmarek, Agnieszka Tafelska-Kaczmarek, Katarzyna Roszek, Joanna Czarnecka, Beata Jędrzejewska, Katarzyna Zblewska

**Affiliations:** 1 Faculty of Chemistry, Nicolaus Copernicus University in Torun, 87-100 Toruń, Poland; tafel@chem.umk.pl (A.T.-K.); k.zblewska@umk.pl (K.Z.); 2 Department of Biochemistry, Faculty of Biological and Veterinary Sciences, Nicolaus Copernicus University in Torun, 87-100 Toruń, Poland; kroszek@umk.pl (K.R.); j_czar@umk.pl (J.C.); 3 Faculty of Chemical Technology and Engineering, Bydgoszcz University of Science and Technology, Seminaryjna 3, 85-326 Bydgoszcz, Poland; beata@pbs.edu.pl

**Keywords:** chitosan modification, fluorescence, heterocyclic aromatic compounds, UV–C irradiation, cytotoxicity

## Abstract

Chitosan is a valuable, functional, and biodegradable polysaccharide that can be modified to expand its applications. This work aimed to obtain chitosan derivatives with fluorescent properties. Three heterocyclic aromatic dyes (based on benzimidazole, benzoxazole, and benzothiazole) were synthesized and used for the chemical modification of chitosan. Emission spectroscopy revealed the strong fluorescent properties of the obtained chitosan derivatives even at a low *N*-substitution degree of the dye. The effect of high-energy ultraviolet radiation (UV–C) on modified chitosan samples was studied in solution with UV–Vis spectroscopy and in the solid state with FTIR spectroscopy. Moreover, cytotoxicity towards three different cell types was evaluated to estimate the possibilities of biomedical applications of such fluorescent chitosan-based materials. It was found that the three new derivatives of chitosan were characterized by good resistance to UV–C, which suggests the possibility of using these materials in medicine and various industrial sectors.

## 1. Introduction

In recent years, the search for photosensitive compounds that could be used in biomedical sciences and modern technologies has become an increasing challenge. Such compounds need to be used in photonics, fluorescence imaging, medical diagnostics, or therapy [1,2,3,4,5]. Especially important is the use of light-emitting biomarkers (or molecular probes) for the diagnostics of tumor diseases via fluorescence imaging, which allows for the visualization of infected tissues in the human body in vivo. In addition to detecting inflammatory and disease foci, photosensitive compounds are also used in anti-cancer therapy (so-called photodynamic therapy, PDT). Photosensitive compounds are also widely applied in modern technologies, e.g., as photoresists for optical lithography [6,7], sensitizers for solar cells [8], or initiators in fast photopolymerization processes [9], and recently also in light-controlled radical polymerization [10].

Among many different luminescent compounds with potential applications, heterocyclic systems including, for example, derivatives of pyrrole, pyridine, pyrazolone, imidazoles, or quinoline are the subject of intensive research [11,12,13,14,15,16]. Heterocyclic compounds containing at least two heteroatoms in the rings, which can additionally be linked to other moieties (aromatic, functional groups such as OH, CHO, COOH, NH_2_), are characterized by various chemical and physical properties. 

It is worth emphasizing that many heterocycles containing nitrogen, oxygen, or sulfur atoms show biological activity; therefore, they are commonly used in the production of current drugs [16,17,18,19], although some of them may be toxic [20,21,22]. They can exhibit various therapeutic effects, e.g., anti-cancer, antibacterial, antiviral, analgesic, anti-diabetic or anti-inflammatory actions. It is enough to mention that many commonly available drugs are based on a variety of heterocycles (e.g., penicillin, cephalosporin, reserpine, or morphine).

However, light-sensitive organic compounds (dyes, sensitizers) do not form thin layers, microcapsules, or other shapes that are required in many practical applications. Besides, they are often unstable when exposed to light and undergo photobleaching, losing their valuable properties. Another disadvantage of these compounds is their solubility in organic solvents, which are generally not accepted in medical applications. The solution to these problems is the introduction of these compounds into the polymer matrix in the process of chemical or physical modification, e.g., by immobilization on the polymer surface as well as preparation of photosensitizer–polymer nanoparticles [23,24,25,26,27,28]. When such a system is intended for biomedicine, it is very important to choose a type of polymer that cannot interact with the human body or cause allergies.

Such conditions are met by biopolymers, and chitosan (CS) is a particularly good candidate for a protective coating of photosensitive or bioactive compounds or as a drug carrier [29,30]. This readily available polysaccharide is the deacetylation product of chitin obtained from shellfish shells or fungal and yeast cell walls.

It is a polysaccharide which, thanks to its chemical structure, has unique properties, namely biodegradability, biocompatibility, nontoxicity, solubility in slightly acidic media, antimicrobial activity, fluid absorptivity, and the possibility of easy modification owing to the presence of hydroxyl and amine functional groups [30,31]. These advantages are the reason for the great interest in this biopolymer. Because it is a material derived from renewable sources, it is therefore referred to as an environmentally friendly material. 

The physical and chemical properties of chitosan, dependent mainly on the molecular weight and degree of deacetylation, are well-known thanks to the intensive research of the last few years, which simultaneously has contributed to its wide application [30,31,32]. Chitosan, which is a copolymer of β-(1→4)–linked D–glucosamine and *N*–acetyl–D–glucosamine, forms a helical flexible structure with repeating pyranose units. 

In solutions, it can exist in various conformations but its ability to form aggregates is low due to the repulsive electrostatic interactions of protonated amine groups in an acidic medium. In the solid state, it crystallizes to a significant extent, creating fibrils and several polymorphic forms. 

Based on a review of the current literature, fluorescent chitosan, due to its unique properties, is the subject of numerous studies [33,34,35,36,37,38,39,40,41,42]. As shown recently, it can be successfully modified with various organic fluorophores, e.g., rhodamine-based dyes [33,34,35], coumarin [36], BODIPY-type compounds, which are dyes consisting of boron difluoride and a dipyrromethene group (e.g., BODIPY containing additional nitro or amino substituents) [37], a derivative of fluorescein [38,39] or substituted naphthalimides [40].

Rhodamine derivative (tetramethylrhodamine isothiocyanate) covalently bound to chitosan was proposed as a material allowing for determination of the degradation of scaffolds. This labeled chitosan was biocompatible and promoted the growth of bone cells. The disintegration of the material could be observed for up to 16 weeks in vitro and for up to two weeks in vivo. Thus, this non-invasive method is very promising in tissue engineering [33].

An interesting proposal for the modification of chitosan and its application was presented by Moreau et al. [34]. Fluorescent chitosan obtained by grafting with rhodamine or fluorescein in the form of a hydrogel was loaded with gadolinium chelate. This system is an innovative magnetic resonance contrast agent, the effectiveness of which is based on the combination of magnetic and optical imaging. It is, therefore, a means to improve magnetic resonance imaging (MRI) contrast and precision and therefore facilitates medical diagnostics.

Another work was related to the study of the effects of allocolchicinoid heterocyclic compounds with antitumor activity similar to colchicine, but with lower toxicity [35]. This drug was introduced into fluorescent chitosan labeled with rhodamine and then applied to mice. Fluorescence studies allowed for determination of the accumulation, distribution, metabolism, and excretion of the drug used, which provides valuable information about its therapeutic effect.

A fluorescent biopolymer such as coumarin-chitosan [36] or BODIPY-chitosan [37] can be used to detect and remove heavy metals from contaminated water or wastewater. For example, an effective quenching of the fluorescence of BODIPY-chitosan by iron ions was observed, and more than 60% of these impurities were removed from aqueous solutions by adsorption. In the case of the coumarin-chitosan sensor, Fe^2+^ adsorption capacity was 63.2 mg/g and the detection limit was 9.6 μM.

Chitosan modified with fluorescein 5-isothiocyanate can be applied in the studies of polymers with the use of ultracentrifuges, in particular for determining the sedimentation coefficient and hydrodynamic parameters of macromolecules [38].

It was also proved that low molecular weight chitosan (an oligomer with a molecular weight of approx. 1000 Da) coupled to fluorescein isothiocyanate is an effective sensor for the selective determination of copper ions in aqueous solutions [39].

The use of bromonaphthalimide as fluorophore allows for further chemical modification, in particular the exchange of bromine atoms with dimethylamine, piperidine, and methoxide, which extends the range of chitosan derivatives intended for biological markers [40].

Apart from the chemical substitution of organic dyes, there are descriptions of obtaining fluorescent chitosan without the use of photosensitive modifiers. An example is a work by Padilha et al. [41], in which fluorescent chitosan nanoparticles were obtained in the process of hydrothermal treatment. Simultaneous use of a hydrochloric acid solution led to the depolymerization of the macromolecules. An anti-cancer compound, methotrexate, was successfully introduced into the chitosan nanoparticles obtained in this process. The fluorescence measurement allowed for in-situ tracking of the drug delivery systems [41].

Moreover, as Geng et al. showed [42], cross-linking of chitosan with glutaraldehyde makes it possible to obtain a fluorescent hydrogel, which is recommended for the detection of trace amounts of toxic heavy metal ions in water. This procedure is based on the observation that mercury ions effectively quench the fluorescence of the cross-linked chitosan.

Our work aimed to chemically modify chitosan with organic photosensitive heterocyclic compounds (aromatic derivatives of benzimidazole, benzothiazole, and benzoxazole—all containing a substituted reactive aldehyde group), leading to obtaining a new material with specific photochemical and biological properties.

The biopolymer in which the particles of the modifying compound were introduced provided the system with properties typical of macromolecular compounds; in particular, the ability to form thin films, good mechanical properties, resistance to environmental factors, and low density. 

## 2. Materials and Methods

### 2.1. Materials

Chitosan with a molecular weight of 50,000 Da and a deacetylation degree of 87% (determined by solid-state ^13^C-NMR) was purchased from Sigma-Aldrich. High purity grade reactants (benzimidazole, benzothiazole, benzoxazole, and terephthalaldehyde) were also supplied by Sigma-Aldrich. Solvents (acetic acid, acetic anhydride, acetone, and methanol) were purchased from POCH™ (Avantor™ Performance Materials, Gliwice, Poland). All chemicals were used without further purification.

### 2.2. Synthesis of Functionalized Heterocyclic Dyes

**Scheme 1:**
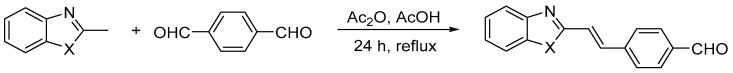
The synthesis of heterocyclic aromatic dyes [43]. Where X = NH, O, or S.

**Figure 1:**
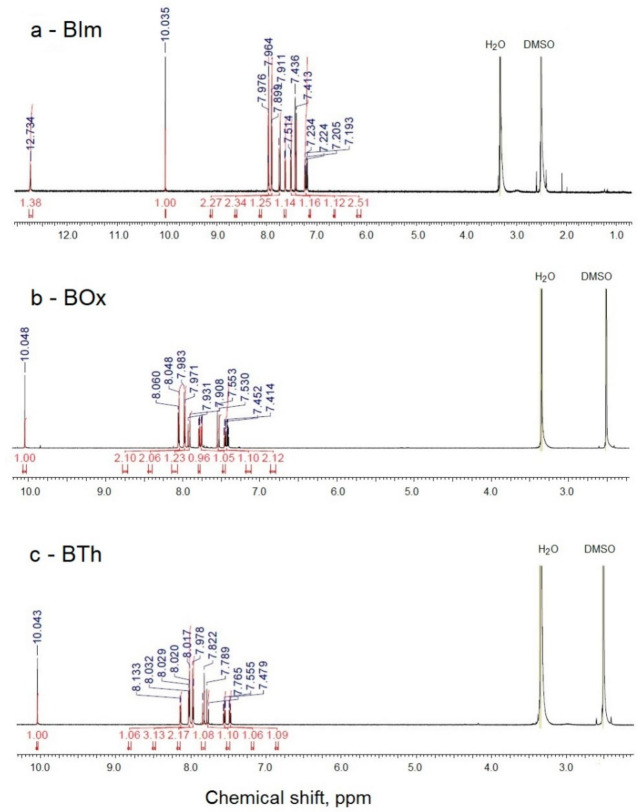
^1^H NMR spectra of dyes BIm (**a**), BOx (**b**), and BTh (**c**).

Three heterocyclic compounds containing aldehyde groups were obtained from the synthesis of 2-methylbenzimidazole, 2-methylbenzoxazole, and 2-methylbenzothiazole with terephthalaldehyde (benzene-1,4-dicarboxaldehyde) in the presence of acetic acid and acetic anhydride [43]. After conditions were optimized, the reaction was carried out at reflux for 24 h. A shorter time (6 h) resulted in lower reaction efficiency, while a longer time (48 h) did not improve the yield. The general scheme of the reactions is shown below (Scheme 1). The reaction yields, given in Table 1, were in the range of 35–48%. The chemical structure of the BIm, BOx, and BTh compounds was confirmed by spectroscopic methods. All dyes were soluble in methanol. ^1^H NMR spectra of the three obtained dyes are shown in Figure 1.

#### 2.2.1. Synthesis of Trans-2-[2-(4-formylphenyl)ethenyl]benzimidazole (X=NH)—BIm

A mixture of 2-methylbenzimidazole (0.67 g, 5 mmol), terephthalaldehyde (0.67 g, 5 mmol), acetic anhydride (1.5 mL) and acetic acid (0.75 mL) was stirred at reflux for 24 h. After cooling to RT, concentrated hydrochloric acid (7.5 mL) was added and the mixture was stirred for 1 h. After filtration, the filtrate was treated with 30% aq. solution of sodium hydroxide (15 mL) and the precipitate was filtered out and dried. The product was purified by column chromatography (petroleum ether/ethyl acetate 1:1) on silica gel. A yellow solid was obtained at 35% yield, 0.43 g mass, mp 177–179 °C, lit. 178–180 °C [43]. ^1^H NMR (700 MHz, DMSO-*d*_6_) δ (ppm): 7.18–7.21 (m, 1H), 7.22–7.25 (m, 1H), 7.42 (d, *J* = 16.1 Hz, 1H, CH=), 7.52 (d, *J* = 7.7 Hz, 1H), 7.63 (d, *J* = 7.7 Hz, 1H), 7.74 (d, *J* = 16.1 Hz, 1H, CH=), 7.91 (d, *J* = 8.4 Hz, 2H), 7.97 (d, *J* = 8.4 Hz, 2H), 10.04 (s, 1H, CHO), 12.73 (s, 1H, NH). ^13^C NMR (75 MHz, DMSO-*d*_6_) δ (ppm): 121.34 (CH), 128.02 (2xCH), 130.54 (2xCH), 133.29 (CH), 136.27 (C), 142.04 (C), 150.77 (C), 192.92 (CHO). IR neat (cm^−1^): 3321, 3052, 2835, 2740, 1692, 1598, 1415, 1306, 1206, 1167, 970, 801, 750, 491, 439. 

#### 2.2.2. Synthesis of p-[Trans-2-(benzoxazol-2-yl)ethenyl]benzaldehyde (X=O)—BOx

The product was recrystallized from ethanol as a yellow solid with 0.49 g mass, 39% yield, mp 177–179 °C, lit. 178.4–179.6 °C [43]. ^1^H NMR (700 MHz, DMSO-*d*_6_) δ (ppm): 7.40 = 7.43 (m, 1H), 7.44–7.47 (m, 1H), 7.54 (d, *J* = 16.1 Hz, 1H, CH=), 7.76 (d, *J* = 8.4 Hz, 1H), 7.79 (d, *J* = 8.4 Hz, 1H), 7.92 (d, *J* = 16.1 Hz, 1H, CH=), 7.98 (d, *J* = 8.4 Hz, 2H), 8.05 (d, *J* = 8.4 Hz, 2H), 10.05 (s, 1H, CHO). ^13^C NMR (175 MHz, DMSO-*d*_6_) δ (ppm): 111.18 (CH), 117.41 (CH), 120.33 (CH), 125.39 (CH), 126.38 (CH), 128.96 (2xCH), 130.41 (2xCH), 137.08 (C), 138.51 (CH), 140.99 (C), 142.17 (C), 150.40 (C), 162.41 (C), 192.99 (CHO). IR neat (cm^−1^): 3051, 2818, 2732, 1695, 1598, 1531, 1450, 1162, 967, 932, 812, 798, 725, 505, 434.

#### 2.2.3. Synthesis of p-[Trans-2-(benzthiazol-2-yl)ethenyl]benzaldehyde (X=S)—BTh 

The product was recrystallized from ethanol as a yellow solid with 0.64 g mass, 48% yield, mp 159–160 °C, lit. 159.2–159.7 °C [43]. ^1^H NMR (700 MHz, DMSO-*d*_6_) δ (ppm): 7.47–7.49 (m, 1H), 7.54–7,57 (m, 1H), 7.78 (d, *J* = 16.8 Hz, 1H, CH=), 7.83 (d, *J* = 16.8 Hz, 1H, CH=), 7.97 (d, *J* = 7.4 Hz, 2H), 8.02 (d, *J* = 7.4 Hz, 2H), 8.03 (d, *J* = 7.7 Hz, 1H), 8.14 (d, *J* = 7.7 Hz, 1H), 10.04 (s, 1H, CHO). ^13^C NMR (75 MHz, DMSO-*d*_6_) δ (ppm): 122.73 (CH), 123.22 (CH), 125.26 (CH), 126.22 (CH), 127.13 (CH), 128.68 (2xCH), 130.41 (2xCH), 134.74 (C), 136.32 (CH), 136.68 (C), 141.38 (C), 155.86 (C), 166.27 (C), 193.00 (CHO). IR neat (cm^−1^): 3049, 2842, 2745, 1691, 1598, 1567, 1478, 1306, 1209, 1162, 940, 806, 757, 724, 463, 432.

### 2.3. Synthesis of Chitosan Modified with Dyes and Film Preparation

**Scheme 2:**
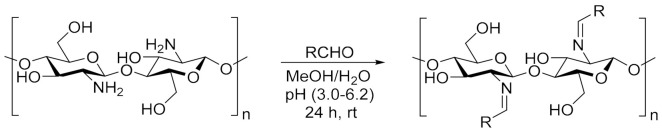
The reaction of chitosan with heterocyclic aldehyde dyes [44]. Abbreviation of obtained specimens: CS-BIm, CS-BOx, CS-BTh. Where RCHO is BIm, BOx, or BTh substituent.

A 2% solution of chitosan (in 1% CH_3_COOH) at a volume of 20 mL was mixed with a 1% methanolic solution of dye (10 mL). The synthesis was carried out according to the described procedure in [44] (Scheme 2). The combined solutions were vigorously stirred with a magnetic stirrer for 24 h in darkness at room temperature. Due to the high volatility of methanol, the system was equipped with a reflux condenser. The solutions were then centrifuged. The content of the added dye was 2–5 wt.%. relative to chitosan. Films of modified chitosan were obtained by pouring the solution into leveled Petri dishes and evaporating the solvent. After thorough drying, the films were gently removed from the substrate. To remove residual acetic acid, samples were washed with 0.1 M NaOH solution followed by distilled water, until completely neutralized. Then, the films were dried to a constant weight in a vacuum dryer at room temperature. During the entire process of obtaining and drying samples, they were protected against light. For spectroscopic tests, the modified polysaccharide solution was poured directly onto the CaF_2_ spectrophotometric windows.

### 2.4. Spectroscopic Analysis (FTIR, UV–Vis, NMR)

Infrared spectra of samples placed on CaF_2_ windows were recorded using a Thermo Scientific™ Nicolet™ iS5 FTIR Spectrometer (Waltham, PA, USA) in transmission mode. The resolution was 4 cm^−1^. Thirty-two scans were collected for each sample. The elaboration of the results and the processing of the spectra were carried out with Thermo Scientific OMNIC™ software (version 9.11.727, USA).

UV–Vis spectra in the range of 200–800 nm were recorded with a UV-PC1600 Shimadzu spectrophotometer Kyoto, Japan). The concentration of dyes in the solutions was 4.5–5.8 × 10^−5^ M. 

The relative (percentage) changes in absorbance, resulting from UV exposure, were calculated for comparison of the degradation degrees of the different samples (Equation (1)):

(1) $$\mathrm{Degradation}\;\mathrm{degree}\;\left(\%\right)=\frac{A_{0}-A_{t}}{A_{0}}\times 100\%$$

where A_0_ and A_t_ are the absorbances at λ_max_ before and after *t* time of irradiation, respectively.

^1^H and ^13^C NMR spectra of samples in DMSO-*d*_6_ or solid-state (^13^C CP-MAS) were measured on a Bruker Avance III 700 MHz (Bruker, MA, USA). The number of scans was 500–2000.

The degree of *N*-substitution (DS) was calculated using NMR spectroscopy according to the methodology proposed by Gonil [44] and Jatunov et al. [45].

### 2.5. Fluorescence

The fluorescence spectra were measured with a RF-5001PC fluorescence spectrometer (Shimadzu, Kyoto, Japan) at room temperature. Excitation maximum was experimentally established at 425 nm and the emission spectra were obtained in a range of 200–800 nm. All samples were prepared as acetic acid/methanol solutions.

### 2.6. Photochemical Stability

Studied samples were irradiated in an air atmosphere at room temperature with a TUV-30W low-pressure mercury vapor lamp (Philips, Holland), emitting a wavelength of 254 nm. The radiated intensity was measured with IL 1400A Radiometer (International Light, Peabody, MA, USA). At the sample level, it was 4.8 mW/cm^2^. The irradiated samples were monitored spectroscopically at specific time intervals. The maximum exposure time for solutions was 2 h and for solid films 12 h. 

### 2.7. In Vitro Cell Culture 

Human dermal fibroblasts (HDF, commercially available, ScienCell Research Laboratories, Carlsbad, CA, USA) were grown in a DMEM-LG (Dulbecco’s Modified Eagle’s Medium, Low Glucose) medium containing 10% FBS (Fetal Bovine Serum) and 1% penicillin/streptomycin solution at 37 °C in a humidified atmosphere with 5% CO_2_.

Human lung epithelial cells (A549 cell line) were purchased from the ATCC collection. Cells were grown as a monolayer in Ham’s F-12 medium containing 2 mM glutamine, 10% fetal bovine serum (FBS), and 1% penicillin/streptomycin at 37 °C in a humidified atmosphere with 5% CO_2_.

Mouse osteoblastic cells (MC3T3) were purchased from Sigma-Aldrich, Darmstadt, Germany. Cells were grown in a MEM (Minimum Essential Medium) containing 2 mM glutamine, 10% fetal bovine serum (FBS), and 1% penicillin/streptomycin at 37 °C in a humidified atmosphere with 5% CO_2_.

### 2.8. Cytotoxicity Evaluation

Prepared specimens (liquids) were pipetted onto the wells of a 96-well plate and left to dry. After 72 h drying under sterile conditions, the layers were washed with 0.1 M NaOH for neutralization, and subsequently with PBS (Phosphate Buffered Saline). During the entire procedure, the samples were protected from light. Subsequently, 0.2 mL of the culture medium was added to half of the plate and incubated for 24 h to obtain conditioned media. These media were used in indirect toxicity tests (according to ISO 10993 standard) and cells were subjected to MTT (3-(4,5-dimethylthiazol-2-yl)-2,5-diphenyltetrazolium bromide) and NRU (Neutral Red Uptake) viability assays. On the other half of the plate, cells were seeded on prepared layers at a density of approximately 3 × 10^4^ cells/well in 0.2 mL medium and cultured for 24 h. To determine cell adhesion to specimens (direct toxicity test), the media with floating cells were collected, centrifuged, stained with trypan blue and counted. 

The MTT test, based on the ability to reduce MTT by mitochondrial dehydrogenases, was performed in triplicate to assess the cell metabolic activity (viability). Five hundred µL of MTT (1 mg/mL; Sigma-Aldrich, Darmstadt, Germany) solution in a suitable culture medium without phenol red was added to each well. After 30 min of incubation at 37 °C in a humidified atmosphere with 5% CO_2_, the solution was aspirated, 500 μL of dimethyl sulfoxide (DMSO; 100% *v/v*; Sigma Aldrich, Darmstadt, Germany) was added to each well and the plates were shaken for 10 min. The absorbance was measured at the wavelength of 570 nm with subtraction of the 630 nm background, using a microplate reader (Synergy HT; BioTek, Winooski, VT, USA). The number of viable cells was calculated relative to the control cells growing directly on the well surface.

The NRU test is based on the neutral red uptake by living cell lysosomes. Aliquots of 0.033% neutral red solution were added to the growing cells and incubated for 90 min. The solution was then aspirated and cells were washed gently with pre-warmed PBS. The accumulated dye was dissolved in 1% acetic acid in 50% ethanol. The absorbance was measured at the wavelength of 540 nm using a microplate reader. The number of viable cells was calculated relative to the control cells growing directly on the well surface.

### 2.9. Statistical Analyses

All experiments were performed in at least triplicate. The mean and standard deviation were calculated for the obtained values. Differences between the test groups were calculated with the Kruskal–Wallis test using PAST 4.02 software (Hammer et al. PAST: Paleontological Statistics Software Package for Education and Data Analysis). The statistical significance of the differences is marked on the graphs with asterisks (* for *p* ≤ 0.05, ** for *p* ≤ 0.01, *** for *p* ≤ 0.001).

## 3. Results and Discussion

### 3.1. Synthesis and Sample Characterization 

Three aromatic heterocyclic dyes with a reactive aldehyde group, the synthesis of which is described in detail in the experimental part, were obtained with moderate yields not exceeding 50%. These compounds had a similar skeleton structure with delocalized π electrons but they differed in the heteroatoms (N, O, S) that existed in the heterocyclic ring (apart from one nitrogen atom). The chemical structure of these compounds was confirmed by spectroscopic methods. The IR spectra (Figure S1) show absorption bands characteristic of both heterocyclic and aromatic compounds (2700–3100 cm^−1^ due to C-H stretching vibrations), and an intense carbonyl band (1690–1695 cm^−1^). The region of 500–1700 cm^−1^ is especially rich in strong, multiple bands. In particular, there may be mentioned ring vibrations and overlapping C=C in-plane at (1400–1600 cm^−1^), CH deformation at (700–900 cm^−1^), and CH=CH in out-of-plane mode at (700–800 cm^−1^).

The coupling of heterocyclic compounds to chitosan is possible due to the reaction of the primary amino groups of CS with the aldehyde moieties of the dyes (Scheme 2). The advantage of the reaction carried out is that it takes place in mild conditions at room temperature and in an air atmosphere. In addition to the introduction of chromophore groups (aromatic and heterocyclic rings coupled with a double bond), the synthesis leads to the formation of imine groups (Schiff base) in polysaccharide chains, which increases the chemical activity of the modified system. 

Difficulties in obtaining a higher degree of conversion were probably due to the cationic nature of this polysaccharide, because of protonated amino groups in the acidic environment in which the reaction was carried out. Moreover, it must be remembered that the properties of chitosan, including chemical reactivity, are not only dependent on the chemical structure of the chain, molecular weight, and the degree of deacetylation; other important factors influencing the course of the reaction may include intra- and inter-chain hydrogen bonds, variable conformations, secondary structure, ionic strength, and distribution of acetyl groups [30,31].

**Figure 2:**
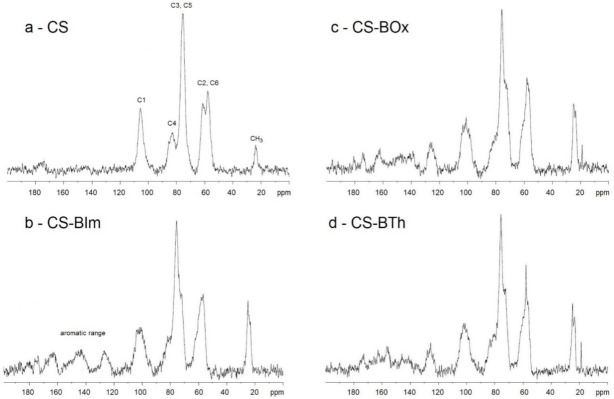
Solid-state ^13^C-NMR spectra of CS (**a**), CS-BIm (**b**), CS-BOx (**c**), and CS-BTh (**d**).

The actual degree of *N*-substitution (DS) can be calculated from ^1^H-NMR spectra [44], because the signals of the protons of the glycosidic rings (H2-H6) from chitosan do not overlap the H signals of the aromatic rings of dyes. However, as Jatunov et al. recommend [45], more reliable results can be obtained from solid-state NMR analysis. This method is advantageous if the chitosan derivatives have low solubility in deuterated solvents. Solid-state ^13^C-NMR spectra of chitosans modified with dyes are presented in Figure 2. The DS was 2.9, 2.7, and 2.4% in CS-BIm, CS-BOx, CS-BTh, respectively. 

The thin films produced by pouring out the modified chitosan solutions were yellowish and visually homogeneous, without surface defects, which were also not visible in microscopic images. 

### 3.2. Fluorescence

The samples of the starting heterocyclic compounds and the modified chitosan exhibited visible fluorescence under UV light (Figure S2); therefore, emission spectroscopy was used.

**Figure 3:**
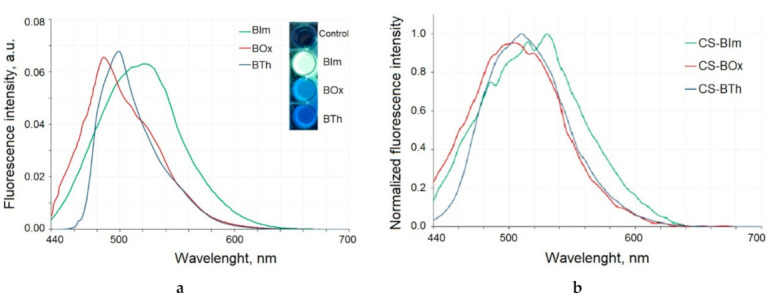
The emission spectra of the dyes excited with 425 nm light (**a**). Inset: the fluorescent properties of dyes excited with UV light (365 nm). Fluorescence spectra of modified chitosan samples (**b**).

In the first step, the fluorescence of the dyes used for chitosan modification was measured (Figure 3a). The excitation maxima were determined to be 426 nm, 429 nm, and 422 nm for BIm, BTh, and BOx, respectively. To standardize the procedure, dyes and modified chitosan samples were excited at the same wavelength of 425 nm for further experiments. All dyes were characterized by strong fluorescence with maxima at a different wavelength: 520 nm (BIm), 485 nm (BOx), and 500 nm (BTh). 

The chemical modification of chitosan with heterocyclic aromatic dyes gives it emission properties as a result of excitation with the 425 nm wave (Figure 3b). The maxima of the emission bands were slightly shifted towards longer waves when compared to dyes alone: in the CS-BIm spectrum it was 530 nm, in CS-Box 505 nm, and in CS-BTh 510 nm. Dye-modified chitosans with fluorescent properties have many advantages because they are easy to track and their biodegradation can be observed as a decrease in fluorescence intensity.

### 3.3. Photochemical Stability

The photochemical stability of modified chitosan was studied both in solution and in solid state. UV-C radiation with a length of 254.6 nm is high-energy radiation capable of breaking chemical bonds in organic compounds. The energy of 1 einstein (i.e., 1 mole of photons) is 470.7 kJ. 

It should be noted that this type of electromagnetic radiation is commonly used to disinfect hospital rooms and sterilize medical accessories, because it effectively removes viruses, bacteria, and fungi, and degrades the DNA or RNA of microorganisms.

#### 3.3.1. UV–Vis Spectroscopy of Solutions

**Figure 4:**
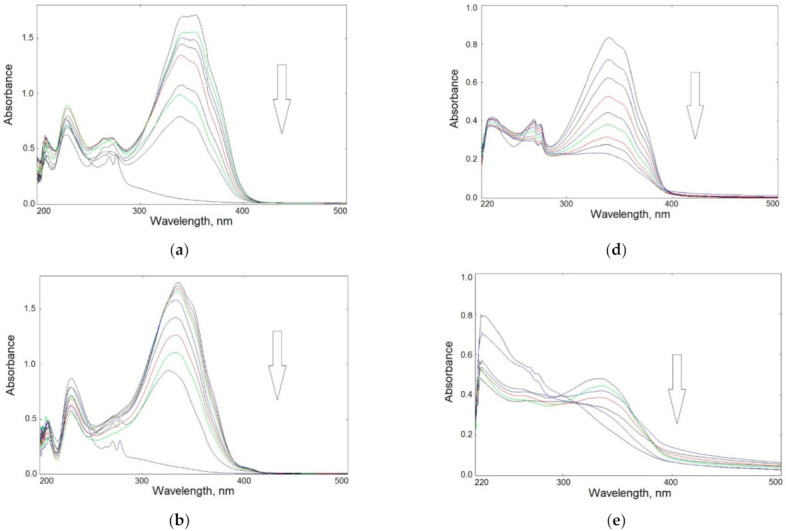

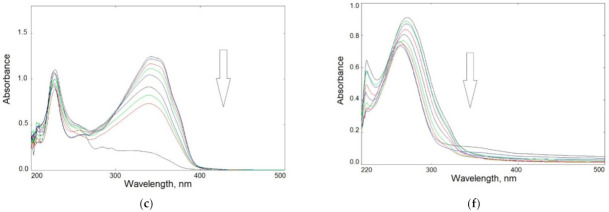
Changes in UV–Vis spectra of UV-irradiated samples in solution: left panel—BIm (**a**), BOx (**b**), and BTh (**c**) in methanol up to 60 min; right panel—CS-BIm (**d**), CS-BOx (**e**), and CS-BTh (**f**) in acetic acid solution up to 120 min (the arrows show the direction of absorbance changes).

First, the effect of UV-C radiation on the obtained samples in solutions was checked. Figure 4a–c shows changes in the UV-Vis spectra of BIm, BOx, and BTh in methanol solutions. The dyes were characterized by high molar extinction coefficients (ε). The ε values of BIm and Box were very close (3.5337 × 10^4^ and 3.6129 × 10^4^ M^−1^·cm^−1^), and this value was only slightly lower for BTh (2.7457 × 10^4^ M^−1^·cm^−1^).

The main absorption band appeared in the 300–400 nm range. The BIm spectrum had a double maximum at 342 and 354 nm; in the BOx and BTh spectra λ_max_ appeared at 335 and 340 nm, respectively. These bands can be attributed to combined π→π* transition in aromatic rings and n→π* in heteroatoms. Chromophore loss, observed as a decrease in absorbance, is a typical photobleaching process.

The disappearance of absorption bands during irradiation indicated rapid photolysis of the dyes: their complete decomposition was observed within about 50–60 min of exposure. 

As recently reported, the main photochemical reactions in heterocyclic compounds are heteroatom isomerization, hydrogen atom transfer, and electrocyclization.^47^ However, various non-absorbing products may be formed in the tested compounds because radiation with a wavelength of 254 nm carries enough energy to break the chemical bonds in heterocyclic rings. In addition to direct photolysis, during which active radicals are formed, secondary processes may take place, i.e., recombination of radicals, an addition to double bonds, or reactions with an aldehyde group. At the same time, it should be remembered that the reactions take place in an air atmosphere, so oxidation reactions will compete with them, yielding oxygen-enriched products (e.g., containing peroxides, hydroxyl, and carbonyl groups). The hydroperoxides that may be formed are the source of hydroxyl radicals which are known as highly reactive species contributing to further secondary reactions.

Absorption spectra of modified CS were significantly altered compared to those of the dyes alone. The maximum absorption of CS-BIm was slightly shifted (337 nm), that of CS-BOx remained at the same wavelength (335 nm), while the highest hypsochromic effect was found in CS-BTh (λ_max_ = 273 nm).

Figure 4d–f shows that changes in UV–Vis spectra of irradiated modified chitosans were slower than those in dyes alone. CS-BIm decomposed most quickly (in about 120 min) among these samples, which was related to the detachment and destruction of the dye substituents. In turn, CS-BOx and CS-BTh samples were much more photostable, as evidenced by slight changes in their UV–Vis spectra. Only in the CS-BTh spectra did a new wideband form in the range of 300–400 nm after longer sample irradiation times (>1 h).

It should be added that changes in the UV–Vis spectra of unmodified chitosan in the range of 250–400 nm were systematic and rather slow (Figure S3). The decrease in absorbance during UV-irradiation of the sample indicated CS photodecomposition connected with the decay of internal impurities and defects as well as the decomposition of acetyl groups. This may also mean that the ionic bonds in chitosan acetate were partially destroyed by UV radiation. The acetamide groups were still present in CS irradiated in solution, as evidenced by the clear wide band with a maximum at 285 nm. It is worth noting that although photodegradation of solid-state chitosan is described in the literature [46], there is a lack of information on the behavior of this polysaccharide during UV-irradiation in solution.

Since, as in the remaining CS samples, the range of 200–300 nm covered the absorption bands for heterocyclic substituents, it is difficult to conclusively identify the changes that took place in the chains of this biopolymer. A complete lack of long-wavelength absorption in CS-BIm (above 400 nm) may indicate the photostabilization of CS chains caused by the presence of introduced chromophores.

**Figure 5:**
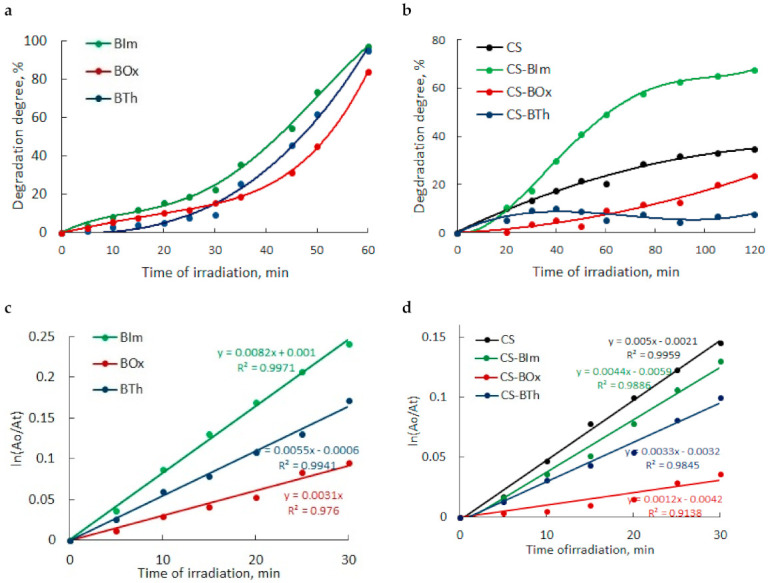
Degree of photodegradation versus irradiation time (expressed as relative absorbance changes) of heterocyclic compounds (**a**) and chitosan samples (**b**) in solution; kinetics of photodegradation of all samples in the initial stage of irradiation up to 30 min (**c**,**d**).

Based on the relative changes in absorbance at the band maximum (Figure 5a,b), the kinetics of photolysis over time were determined for all specimens. For small degrees of degradation (<20%, during the initial stage of irradiation i.e., up to 30 min), a rectilinear dependence of ln(A_o_/A_t_) in the function of exposure time was found (Figure 5c,d). This indicates a pseudo-first-order reaction in all cases. 

The determined slope of the lines indicated that the dye photolysis rate was as follows:

BIm > BTh > BOx

After 45 min, the degradation rate in all samples increased rapidly. Finally, after 1 h of UV-irradiation, all dyes decomposed completely.

As can be seen, the photochemical decomposition of modified chitosan films was slower than that of the dyes themselves. The rate of photodegradation of chitosan samples can be ranked in order:

CS > CS-BIm > CS-BTh > CS-BOx

The degradation degree was lower in CS-BOx and CS-BTh (not exceeding 30%) than that in CS alone. Only CS-BIm was characterized by higher efficiency of this process (about 60% after 80–120 min UV exposure). The observed changes were mainly caused by detachment or destruction of the heterocyclic substituents and only CS-BIm displayed a relatively high content of substitution.

Generally speaking, studies show a photoprotective effect of chitosan, which was also observed in composites of this polysaccharide with squaraine and porphyrazine-type dyes [47,48].

#### 3.3.2. FTIR Spectroscopy of Solid Films 

**Figure 6:**
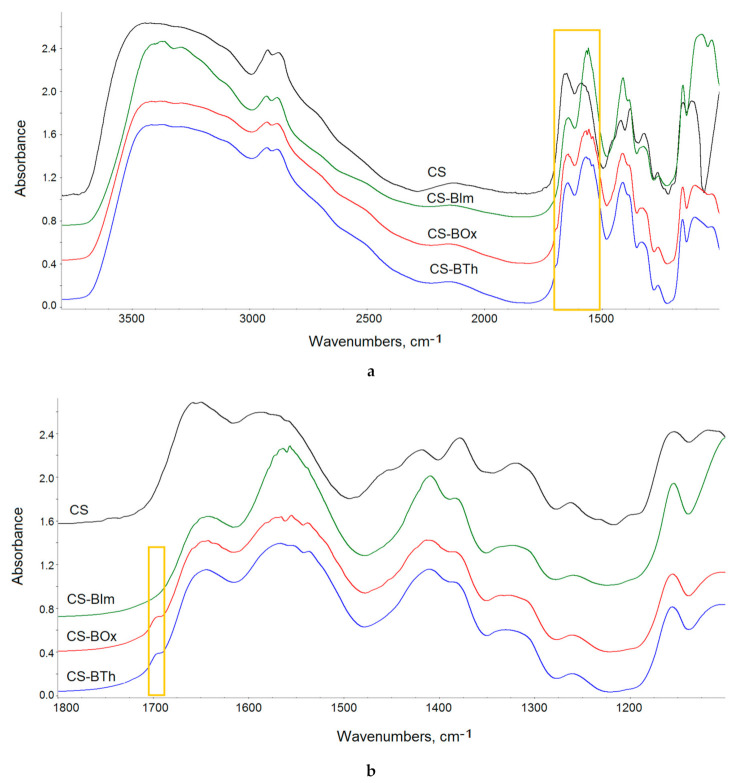

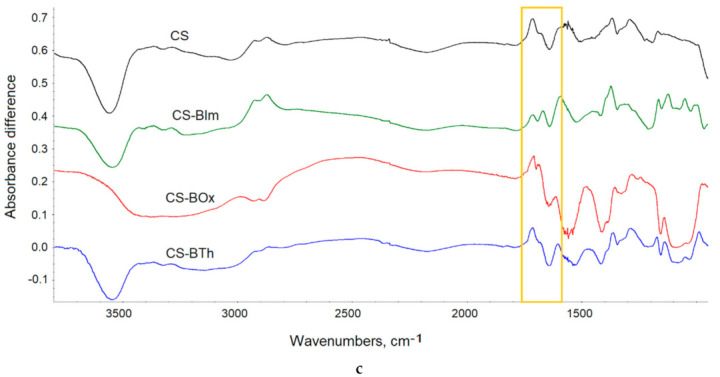
FTIR spectra of CS and its N-derivatives: at full IR range (**a**), at 1100–1800 cm^−1^ range (**b**); difference spectra (**c**) obtained by subtraction of the spectrum of the unirradiated samples from the spectra of 8 h UV-irradiated samples. The yellow rectangles show the bands discussed in the text. In all figures (**a**–**c**), an offset was used to present the spectra.

In the next step, the influence of UV-C radiation on the pure (CS) and modified chitosan films (CS-BIm, CS-BOx, CS-BTh) was examined using infrared spectroscopy Figure 6. Typical chitosan absorption covers several ranges corresponding to the stretching vibrations of OH/NH (broad band at 3000–3600 cm^−1^), methyl/methylene (2800–300 cm^−1^), and carbonyl (1600–1700 cm^−1^) groups. Amine/amide vibrations (N-H and C-N) appear at 1641 (amide I), 1555 (amide II) cm^−1^, and CH_2_ bending at 1412 cm^−1^. Moreover, the fingerprint range is rich in bands characteristic of polysaccharides, among which the band corresponding to the stretching vibrations of the C-O linkages at 1117 cm^−1^ is the most intense [49,50].

Chemical modification insignificantly altered the FTIR spectra of chitosan, because of the low degree of substitution. Both the aromatic and imine bands overlapped with the chitosan absorption bands and were therefore difficult to detect (Figure 6a). However, detailed spectra analysis shows that the ratio between the intensity of amide I and amide II bands changed from 1.01 in CS to 0.735, 0.81, and 0.741 in CS-BIm, CS-BOx, and CS-BTh, respectively (these bands are distinguished by a yellow rectangle in Figure 4a). The relative decrease in the 1555 cm^−1^ band indicated the partial disappearance of NH_2_ groups as a result of chemical modification of chitosan, i.e., formation of a Shift base. In the cases of the CS-BOx and CS-BTh spectra, a low intensive branch occurred at approximately 1692 cm^−1^ (Figure 6b), which can be attributed to C=N and ethenyl (C=C) vibrations in modified CS. Difficulties in detecting the bands characteristic of organic nitrogen-containing compounds in infrared spectra were also signaled by other authors [51].

Collected FTIR spectra showed small changes, which were observed in all exposed samples (Figure S4). The systematic fall in the hydroxyl band proved that weakly and strongly bonded water evolved during UV-irradiation. Simultaneously, the systematic decrease in absorption in the amide region (1500–1700 cm^−1^) and glycosidic bond vibrations (900–1300 cm^−1^) indicated the possibility of substituent abstraction and random breaking in the chitosan backbone. The weak peak due to imine/ethylene bonds completely disappeared at the first irradiation period (1 h), which can be explained by the addition of oxygen or other low molecular species to double bonds. 

In recorded spectra of UV-irradiated samples, practically no new bands were visible, suggesting that photooxidative degradation was irrelevant in these conditions. To precisely analyze the chemical structure, difference spectra were produced by subtraction of the spectrum of the unexposed sample from the spectrum of the same sample after UV irradiation (Figure 6b). Positive changes in the difference spectrum indicated the formation of new bands, while negative changes proved the disappearance, i.e., the decay of the corresponding groups. As can be seen, alongside the decrease in absorbance in the range of hydroxyl/amine (OH/NH) and ether groups (C-O-C), the appearance of weak carbonyl bands was observed. In particular, these bands had maxima at 1715, 1716, 1710, and 1714 cm^−1^ in CS, CS-IBm, CS-BOx, CS-BTh, respectively. These bands can be attributed to formed ketone and aldehyde groups, confirming the photooxidation process. However, the found changes were small (ΔA did not exceed 0.02). 

As shown in previous works on chitosan exposed to UV radiation, the changes in the absorption spectra are caused by decomposition reactions, mainly chain scission resulting in cleavage of glycosidic bonds, dehydroxylation, dehydroxymethylation, and pyranose ring-opening with simultaneous photooxidation, leading to the formation of new carbonyl moieties [52,53]. The mechanism of the photo-oxidative degradation of CS is via free radicals, which was confirmed by the electron spin resonance (ESR) method [52].

In the latest work on the effect of UV radiation (λ > 300 nm) on chitosan, the photodegradation mechanism was re-analyzed and summarized based on new experimental results [53]. The formation of volatile products such as amide and acetamide was detected by solid phase micro extraction and gas chromatography with mass spectrometry (SPME-GC/MS). These products are a result of the abstraction of side groups from the saccharide chains. The ketone groups absorbing at 1730 cm^−1^, monitored by FTIR, were identified as gluconolactone. Moreover, as stated in this study, chitosan exposed to long-term exposure (400 h) was almost completely cross-linked (96%), which further affected the mechanical properties in nanoscale.

Our results for irradiated chitosan differed slightly from those cited above due to a different source of radiation (lamp emitting short-wave high energy radiation). Modified CS samples were characterized by relatively good photoresistance, which indicates the possibility of sterilizing them with bactericidal UV-C radiation. The photostability can be partly explained by the consumption of the absorbed energy during the fluorescence process, instead of a chemical reaction. As can be seen (Figure 3), in the case of the tested systems, emission processes competed with photochemical reactions. Moreover, good photostability can be caused by superficial crosslinking, possibly by the presence of unsaturated bonds in the substituents (N=C, C=C). This protects the deeper specimen layers against the penetration of radicals and oxygen diffusion.

Comparing the course of photochemical reactions of the modified CS in different physical states, it can be concluded that the processes taking place in thin films were much slower and less efficient than in solution. This is related to the low mobility of macroradicals and the slow diffusion of small radicals in solid films, which favors the cage effect. This means that the formed radicals quickly recombined at the point of their creation. In solution, the mobility and diffusion of these active species responsible for photodegradation were much greater, which facilitated contact with other particles. 

### 3.4. Determination of Cell Viability

Cell viability tests (direct and indirect) were performed by seeding human dermal fibroblasts (HDF), lung epithelial cells (A549), and osteoblastic cells (MC3T3) onto studied CS samples and untreated wells with the addition of conditioned media. 

**Figure 7:**
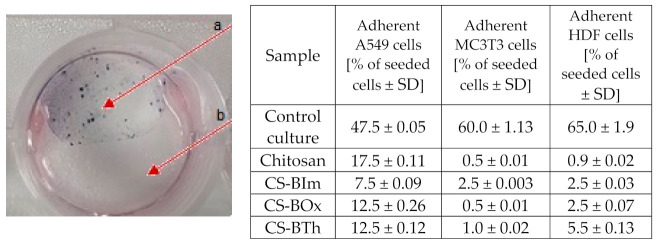
Representative example of a cytotoxicity test on a modified chitosan sample. Arrow *a* shows the violet formazan crystals formed from MTT (see text for details) in viable HDF cells growing on a chitosan-free surface; arrow *b*: cell-free chitosan layer. Table: adhesion test after 24 h culture on different CS samples.

The modified chitosan samples were found not to support cell adhesion, but on the other hand, they were non-toxic. Cells grew adjacent to, but not directly on top of, the samples (Figure 7). The enzymes of the redox-active mitochondria reduced MTT to colored formazan crystals. These crystals dissolved in DMSO, and the color intensity of the resulting solution was directly proportional to the activity of mitochondria and the number of living cells. Interestingly, the materials also showed fluorescence after cell viability tests, which means that under physiological conditions the samples are stable over time, and natural cells and their microenvironment do not disturb the photophysical processes.

The differences between the behavior of virgin CS and its modified forms were due to the changes in surface properties, because attached dyes, as hydrophobic substituents, decreased biopolymer hydrophilicity. Even if this change was minor, it had a visible effect on cell colonization. Quantitative results reflecting cell adhesion ability on CS and different modifications are collected in Figure 7.

**Figure 8:**
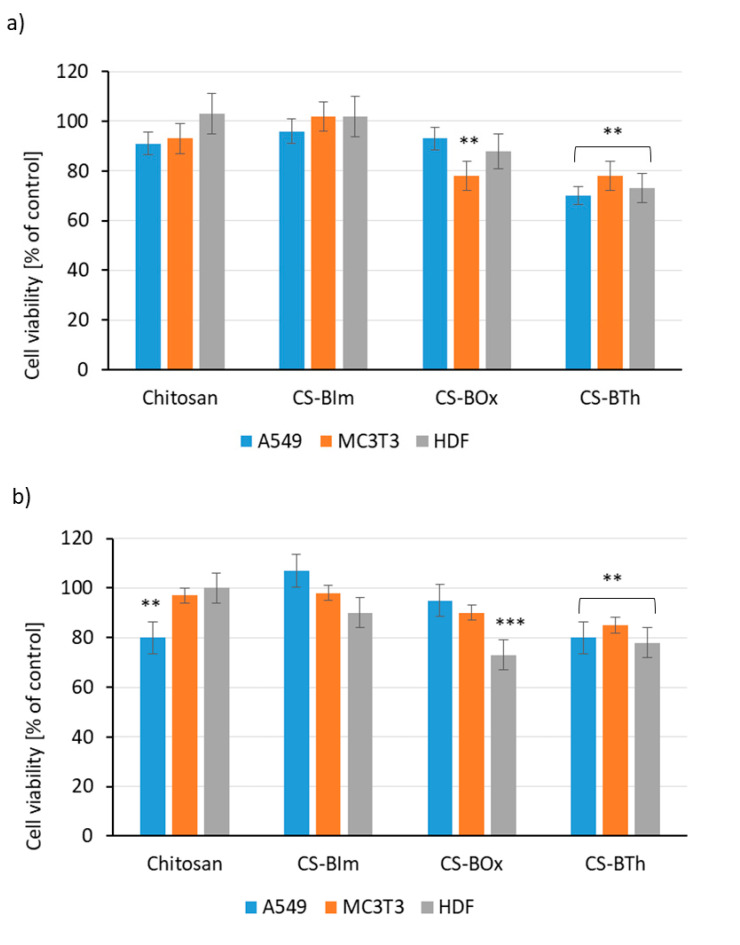
Results of MTT assay (**a**) and NRU test (**b**) for lung epithelial cells (A549), osteoblastic cells (MC3T3) and dermal fibroblasts (HDF). The data are presented as mean ± SD. The statistical significance of the differences is related to control samples and marked on the graphs with asterisks (** for *p* ≤ 0.01, *** for *p* ≤ 0.001).

The results of indirect viability assays for cells growing in conditioned media (after their 24 h incubation with all CS specimens) are presented in Figure 8. A slight decrease in viability (metabolic activity) can be observed for all cell types cultured with CS-BTh-conditioned media (Figure 8a). The results of the NRU test indicate that the chitosan-conditioned medium aggravated viability of A549 cells, while CS-BOx- and CS-BTh-conditioned media decreased viability, mainly of HDFs (Figure 8b). Results of indirect assays indicated that the studied specimens did not release any toxic substances and also did not adsorb vital medium compounds, e.g., growth factors. In other words, they would not reduce the growth and proliferation rate of dermal fibroblasts, lung epithelial cells and osteoblastic cells even in areas adjacent to the sample. 

It can be concluded from the results presented above that CS and dye-modified CS did not create a cell-friendly, biocompatible surface facilitating cell adhesion. Nevertheless, the lack of toxic influence could be also beneficial and allows for claiming CS and dye-modified CS as bio-inert materials potentially applicable in biomedicine. The phenomena of CS-mediated cell adhesion inhibition is surprising given that CS is a natural polymer, reported in the literature as nontoxic, biocompatible, and biodegradable. Therefore, it is one of the most frequently used biomaterials for drug delivery, cell culture, or tissue engineering [54,55]. Modification with heterocyclic compounds was expected to change the biological properties of CS; however, the cell adhesion inhibition was similar in all tested specimens.

On the other hand, chitosan and CS-based materials with dyes were non-toxic directly, which allows for the assumption they do not interact with the cellular microenvironment. Materials with bio-inert properties are also required in the field of biological research. Nevertheless, our preliminary cytotoxicity data indicate that this issue needs further elucidation.

## 4. Conclusions

Three synthesized heterocyclic aromatic compounds were successfully used for the chemical modification of chitosan to obtain fluorescent derivatives. The received materials were film-forming, which is an advantage for a variety of applications.

The dye *N*-substituted chitosan samples exhibited fluorescence properties at 425 nm excitation. Emission at maximum wavelengths of 530, 505, and 510 nm appeared in CS-BIm, CS-BOx, and CS-BTh, respectively. These properties were stable over time and did not change under the influence of human cells and their microenvironment.

The effect of UV irradiation on chitosan derivatives was studied in solution and solid state using UV–Vis and FTIR spectroscopy, respectively. It was found that efficient photobleaching appeared in solutions of initial dyes but this process was slower in corresponding modified chitosan specimens. The photochemical decomposition of studied samples followed the first-order kinetics in the initial stage of irradiation (up to 30 min).

UV irradiation of solid films caused much slower and less efficient reactions than that in solutions. Only slight changes in the chemical structure of the modified chitosan were observed, which indicates good photostability and the protective effect of the dye substituents by the chitosan backbone. This finding makes it possible to use UV radiation for sterilization products based on the proposed materials, if necessary.

CS and dye-modified CS hampered adhesion of lung epithelial cells, osteoblastic cells, and fibroblasts; however, the results of indirect toxicity tests revealed the non-toxic nature of the novel materials. These tests indicate that modified chitosans are bioinert; that is, they will not interact with human tissues, which can be an advantage in such biomedical applications where there is long-term material contact with the human body.

It is also worth emphasizing that, thanks to the presence of C=N and C=C functional groups in macromolecules, the chemical and biological reactivity of CS changed. These unsaturated groups are responsible for material crosslinking; moreover, the imine moieties contribute to an increase in the antibacterial and antifungal activity of chitosan, as Jin and coworkers proved earlier [56].

## Figures and Tables

**Table 1:** The abbreviations, chemical structures, two alternative names, and reaction yields of obtained dyes.

(a) BIm	(b) BOx	(c) BTh
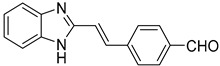	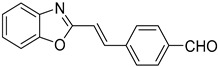	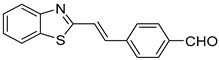
*trans*-2-[2-(4-formylphenyl)ethenyl]benzimi- dazole; 4-[(1*E*)-2-(1*H*-benzimidazol-2-yl)ethenyl]benzaldehyde	*p*-[*trans*-2-(benzoxazol-2-yl)ethenyl]benzaldehyde; 4-[(1*E*)-2-(2-benzoxazolyl) ethenyl]benzaldehyde	*p*-[*trans*-2-(benzthiazol-2-yl)ethenyl]benzaldehyde; 4-[(1*E*)-2-(2-benzothiazolyl) ethenyl]benzaldehyde
35%	39%	48%

## Data Availability

The data supporting the findings of this study are available within the article.

## Supplementary Materials

The following are available online at https://www.mdpi.com/article/10.3390/ma14216429/s1, Figure S1: ATR-FTIR of three modifying compounds: BOx, BIm, and BTh; Figure S2: Fluorescence of chitosan derivatives under 365 nm light: CS-BIm (a), CS-BOx (b), and CS-BTh (c); Figure S3: UV-Vis spectra of unmodified chitosan (2% solution in acetic acid) exposed to UV-C radiation in time up to 120 min; the arrow shows the direction of absorbance changes; Figure S4: Changes in FTIR spectra (offset) of studied films during 0–8 h UV-irradiation: CS-BIm (a), CS-BOx (b) and CS-BTh (c). The spectrum at the top corresponds to the unexposed sample, at the bottom after 8 h UV.
