# Supplementary material for: Fluorescent Chitosan Modified with Heterocyclic Aromatic Dyes
Source: Materials (Basel). 2021 Oct 26;14(21):6429. doi: 10.3390/ma14216429 (PMC8585458; doi:10.3390/ma14216429)
Supplement: Supplementary file 1 [file materials-14-06429-s001.zip › materials-1369058-supplementary.pdf]

Supplementary Materials

Fluorescent Chitosan Modified with Heterocyclic Aromatic Dyes

Halina Kaczmarek 1,*, Agnieszka Tafelska-Kaczmarek 1, Katarzyna Roszek 2, Joanna Czarnecka 2,

Beata Jędrzejewska 3 and Katarzyna Zblewska 1

1 Faculty of Chemistry, Nicolaus Copernicus University in Torun, 87-100 Toruń, Poland;
tafel@chem.umk.pl (A.T.-K.); k.zblewska@umk.pl (K.Z.)

2 Department of Biochemistry, Faculty of Biological and Veterinary Sciences, Nicolaus Copernicus Uni-versity in Torun, 87-100 Toruń, Poland; kroszek@umk.pl (K.R.); j_czar@umk.pl (J.C.)

3 Faculty of Chemical Technology and Engineering, UTP University of Science and Technology Bydgoszcz University of Science and Technology, Seminaryjna 3, 85-326 Bydgoszcz, Poland; beata@pbs.edu.pl

* Correspondence: halina@umk.pl; Tel.: +48-56-6114312

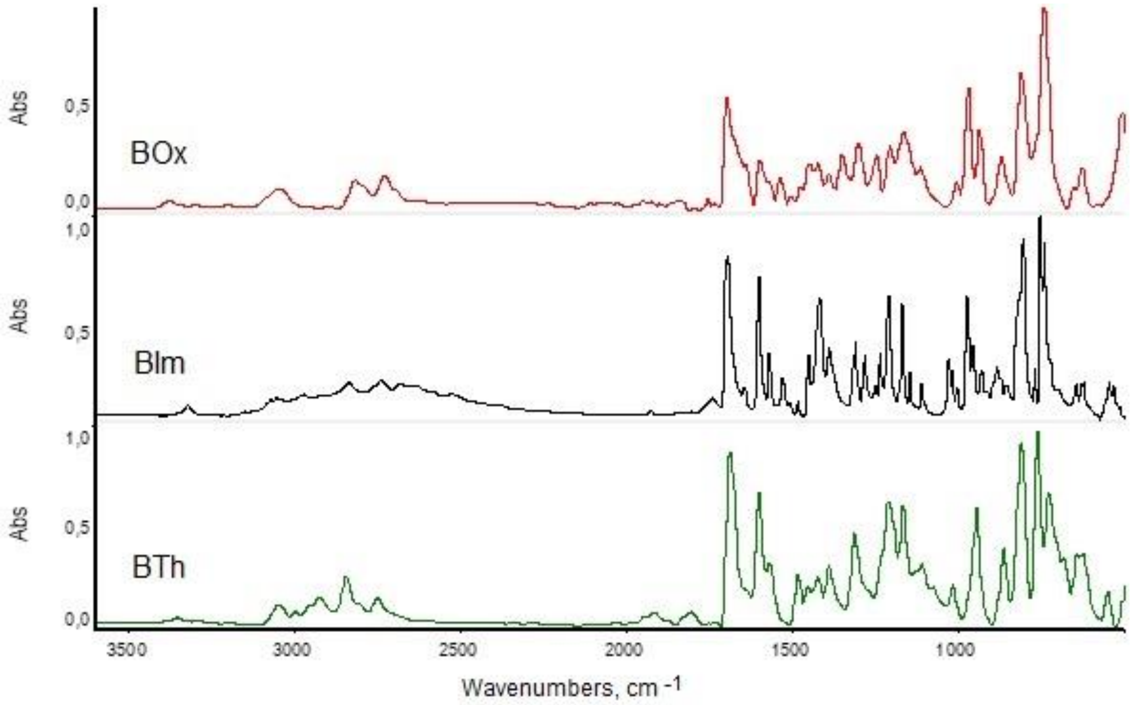

Figure S1. ATR-FTIR of three modifying compounds: BOx, BIm, and BTh.

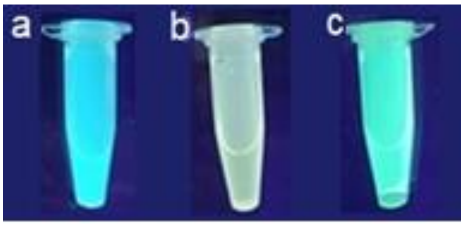

Figure S2. Fluorescence of chitosan derivatives upon 365 nm light: CS- BIm (**a**), CS-BOx (**b**), and CS-BTh (**c**).

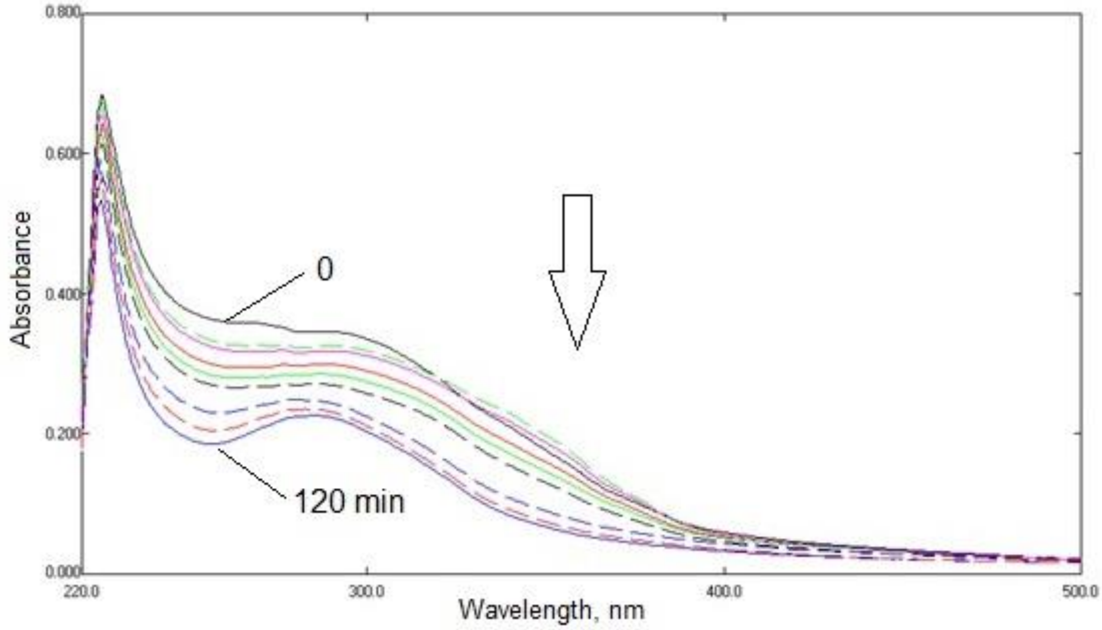

Figure S3. UV-Vis spectra of unmodified chitosan (2% solution in acetic acid) exposed to UV-C radiation in time up to 120 min, the arrow shows the direction of absorbance changes.

a

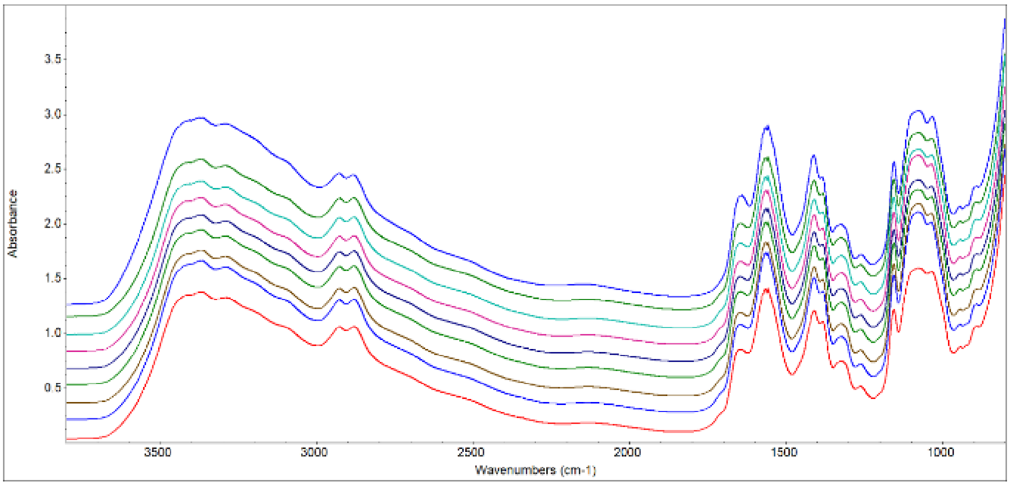

b

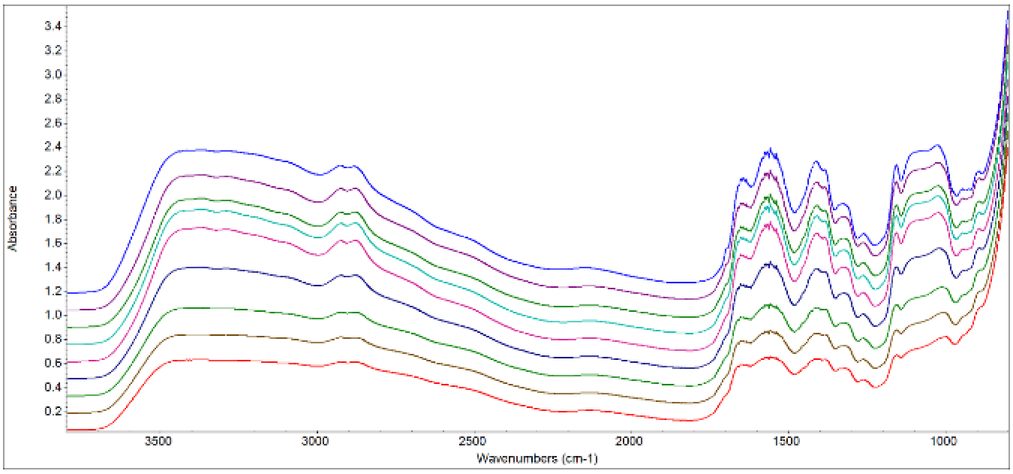

c

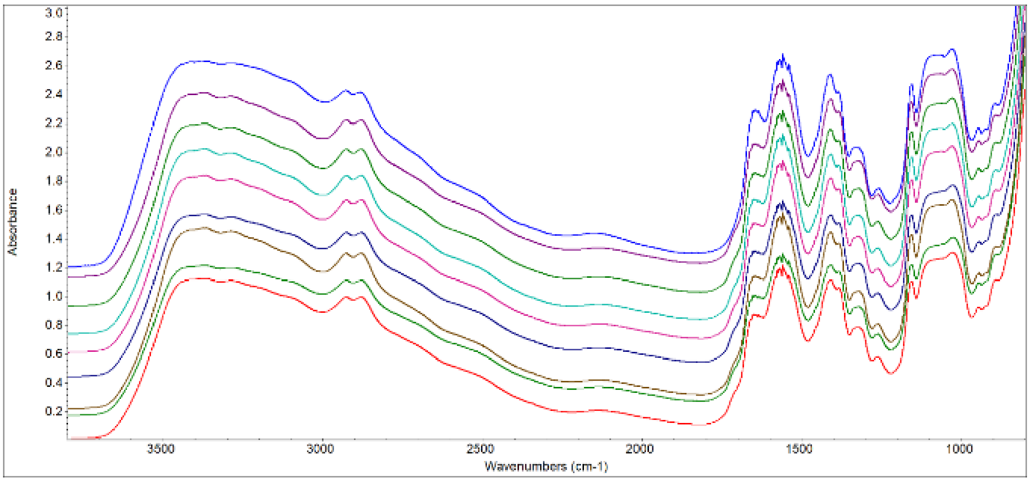

Figure S4. Changes in FTIR spectra (offset) of studied films during 0-8h UV-irradiation: CS-BIm (**a**), CS-BOx (**b**) and CS-BTh (**c**). The spectrum at the top corresponds to the unexposed sample, at the bottom - after 8h UV.
